# COVID-19 and cryo-EM

**DOI:** 10.1107/S2052252520008799

**Published:** 2020-07-02

**Authors:** Sriram Subramaniam

**Affiliations:** a University of British Columbia, Vancouver, BC V6T 1Z3, Canada

**Keywords:** COVID-19, cryo-EM, coronaviruses, SARS-CoV-2, editorial

## Abstract

The application of cryo-EM methods to study SARS-CoV-2 proteins provides a convincing demonstration of the power of cryo-EM in the arsenal of structural biology.

The last few years have seen spectacular growth in the widespread adoption of cryo-electron microscopy (cryo-EM). With the advent of direct electron detectors, the number of depositions in the EM Data Bank (EMDB; https://emdb-empiar.org/) has increased steadily at a rate of ~30% every year since 2014, crossing the 10 000 mark in early 2020.

What may not be widely appreciated is that this extraordinary rate of growth in cryo-EM is not new. The number of depositions in the EM data repository increased on average by ~40% each year between 2004 and 2014, quickly expanding beyond the 52 entries listed in 2004. Besides the sheer increase in numbers of structures being determined using cryo-EM methods, an important aspect to this growth is the application of cryo-EM to determine structures of heterogeneous and dynamic assemblies. Protein complexes of this kind will most likely continue to remain largely intractable to crystallographic approaches that require the generation of ordered two- or three-dimensional crystals.

The rapid progress in the application of cryo-EM methods to study SARS-CoV-2 proteins provides a convincing demonstration of the power of cryo-EM in the arsenal of structural biology. Within a month or so of the availability of the gene sequence, Wrapp *et al.* (2020 ▸) and Walls *et al.* (2020 ▸) were able to obtain the first structures of the soluble part of the trimeric SARS-CoV-2 spike protein. Nearly a dozen groups worldwide have extended these studies in short order to decipher many conformational variants of the spike protein, including structures of native spikes displayed on intact viral membranes. These are major achievements that are also a testament to the speed, quality and biomedical relevance of present day cryo-EM methods. The structures provide important insights into the complexity of spike architecture, especially in the conformational spread of the domain that binds the human ACE2 receptor. These studies, in combination with emerging structural information on the binding sites of various antibodies on the S-protein are fundamentally relevant to the design of effective therapeutics and vaccines against COVID-19.

Before January 2020, of the 84 coronavirus cryo-EM structures deposited in the repository, 82 were of trimeric spike proteins. In contrast, of the 34 entries related to SARS-CoV-2 that have been deposited in 2020, only ~50% are of spike proteins, with the rest coming from entries such as the ORF3a membrane protein, ACE2 protein in complex with the membrane protein BOAT1 and RNA polymerase complexes in different conformational states [Fig. 1 ▸(*a*)]. What is noteworthy about these advances is that protein complexes of this kind are often flexible and conformationally heterogeneous, making them challenging to study by X-ray crystallography. The advent of ‘single particle’ methods such as cryo-EM and XFELs offer the exciting prospect to transcend the description of proteins in terms of static structures, and instead derive conformational landscapes that may provide a much better way to understand their biological function (Ourmazd, 2019 ▸). We are now publishing an increasing number of important papers in this area in **IUCrJ**, and are particularly keen to welcome papers in **IUCrJ** and other IUCr journals that increase understanding of SARS-CoV-2.

The realization of the potential impact of cryo-EM led to the nucleation of several national and regional facilities in different countries to nurture the growth of the cryo-EM field (Subramaniam, 2019 ▸). While these facilities continue to play a key role, we are now seeing growth in the cryo-EM field that is fueled by the increased availability of high-end cryo-EM instrumentation within individual institutions. For this trend to continue, it is critical that the cost of cryo-EM instrumentation necessary for high-resolution structure determination decreases significantly. Funders of biomedical research need to encourage manufacturers to drive costs down to allow the technology to be accessed more readily by the larger biological community.

The critical role played by the EMDB over the last two decades in the dissemination and impact of cryo-EM cannot be underestimated. Established at a time far before the wider recognition of the cryo-EM revolution, the access provided by the repository of structures to both specialists and non-specialists has catalyzed the growth of the field enormously. The recent launch of EMPIAR as an archive where authors are able to deposit raw cryo-EM data has enabled crowd-sourcing of data analysis (https://www.ebi.ac.uk/pdbe/emdb/empiar/). I enthusiastically endorse their appeal to the structural biology community to deposit COVID-19-related raw cryo-EM data in EMPIAR to enable more in-depth analysis following initial publication of the data given the rapid growth in our collective knowledge of this pandemic.

While cryo-EM techniques are being used to glean atomic level information on SARS-CoV-2, it is worth reflecting on the historical context of electron microscopy in the study of these types of viruses. In the 1960s, a young technician named June Almeida made seminal observations in the course of electron microscopic investigations of viruses. Almeida, who trained initially in Canada and subsequently worked at a hospital in London, showed that antibodies derived from infected individuals could be used to coat viruses, thereby enabling their detection in the electron microscope using heavy-atom stains to generate contrast. Among her many contributions to the imaging of viruses such as Rubella and hepatitis B in an electron microscope, one that stood out was the observation of a new kind of virus that she and her colleagues labeled as a ‘coronavirus’ in recognition of the viral surface proteins, which appeared like a crown or ‘corona’ on the membrane.

The resemblance of June Almeida’s image from 1966 [Fig. 1 ▸(*b*)] of a sample referred to as B814 and the iconic recent image of the SARS-2 coronavirus [Fig. 1 ▸(*c*)] produced by scientists at the NIAID laboratories in Montana is remarkable. Looking ahead, a major goal of structural virology will be to bridge the gap between the knowledge of atomic resolution structures of individual viral components and the critical macromolecular interactions that define their assembly along with RNA into an intact, infectious virus. These kinds of integrative structural studies, combined with biological analyses of the processes involved in viral replication will be essential to understand the molecular mechanisms by which SARS-CoV-2 enters cells to cause its devastating effects on human health.

## Figures and Tables

**Figure 1:**
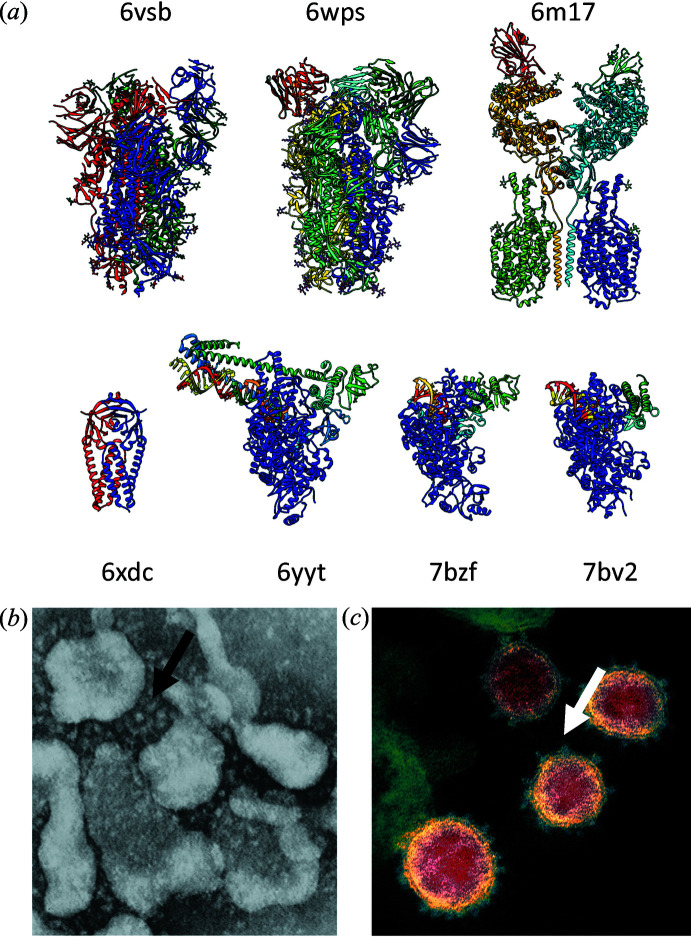
(*a*) A selection of recently reported cryo-EM structures of SARS-CoV-2 proteins including spike proteins, RNA polymerase complexes and membrane proteins in the viral envelope. The PDB identifiers of the structures are indicated. (*b*) Negatively stained transmission electron microscopic image (Almeida & Tyrrell, 1967 ▸) of a novel virus that was named a ‘coronavirus’ by June Almeida in 1966. (*c*) Colorized transmission electron microscope image of negatively stained infectious SARS-CoV-2 particles recorded at NIAID’s Rocky Mountain Laboratories (RML) in Hamilton, Montana. The arrows in panels (*b*) and (*c*) point to the spike proteins on the surface of the virus particles.
